# Hospitals in India

**Published:** 1893-01-07

**Authors:** 


### HOSPITALS IN INDIA.

### I.—THE BENGAL PRESIDENCY.

#### (1) Calcutta Medical Institutions.

In the town and amalgamated area of the suburbs of Calcutta there are eleven medical institutions, which, in 1891, afforded treatment to 245,136 persons, of whom 25,318 were indoor and 219,818 were outdoor patients. Of the grand total, 122,932 were Hindus, an increase of more than seven thousand over the previous year, which is, perhaps, to be explained by the markedly greater liability of Hindus to certain diseases, and especially to cholera; of the others, 77,068 were Mussulmans, 28,096 were Eurasians, 7,713 were Europeans, and the remaining 9,327 were supplied from other classes not specified in the reports. It appears that 1891 was an unusually sickly year, and there was an increase of 956 indoor, and 11,745 outdoor patients, as compared with the previous year; while the total number of deaths throughout the district from the most prevalent diseases rose from 22,241 to 23,650. We do not give the actual death-rates, as they cannot be justly compared with those of 1890, the former having been calculated on the census figures of 1891, while the latter were based on the census of 1881. With respect to the mortality in hospitals, the general death-rate among all the patients treated was 12·7 per cent., against 12·4; but, omitting the deaths from cholera, the rate is reduced to 11·3 per cent., against 11·4 in the previous year. The hospital mortality from cholera coincides very closely with the known mortality among the general population. A feature of the hospital year 1891 was the number of cholera cases, and the cause of the increase is said by authorities to have been the Ardhodoy Jog Festival. We cannot now enter into the wide questions thus opened up, but note with satisfaction that the outcome of the anxiety felt with regard to this disease has been a continuous and remarkable improvement in the sanitary condition of the large hospitals in the Presidency.

Dysentery, diarrhœa, tubercular, respiratory, and rheumatic affections were all more prevalent in 1891 than in 1890, and venereal diseases were said to have increased, more particularly since the abolition of the Contagious Diseases Act, with regard to which we hold very strong opinions, but since the statistics of the disease, as furnished by the hospitals, do not afford much material evidence on the point, we shall not dilate upon it in this instance.

A new classification of surgical operations has been adopted by the Government of India, and the effect of this revision has been to increase the number of major operations at some institutions, and to diminish them at others. It is not possible, therefore, to compare the two years quite justly, but the returns for 1891 show that 2,221 major operations were performed with 107 deaths, or a rate of 4·5 per cent., against 2,080 in 1890 with 88 deaths, or a rate of 3·9. Ten years previously the death-rate stood at 8·6 per cent. Minor operations numbered 20,275 in the year under review, against 19,956 in 1890.

We quote the following passage, which deals with the nursing, verbatim from the Inspector-General's report: "The nurses living and working under the control of Sister Jane Frances, the Sister Superior, and her assistants of the Clewer Sisterhood, have continued to give satisfaction to all the medical officers under whom they are employed in the different medical institutions. Approximately one half are employed at the General Hospital, and the other half at the Medical College Hospital, where new quarters have been built for those employed there. Trained nurses have been employed at the Howrah Hospital, but, owing to want of accommodation, they have not been placed under the Clewer Sisters. At the Campbell Hospital the nursing has been put on a more satisfactory footing, under the orders of his honour the Lieutenant Governor. The efficiency of the nursing at the Presidency General, Medical College, and Eden Hospitals cannot be too highly spoken of. Beside imparting an air of comfort and cheerfulness to the sick-room, it is not too much to say that their services in the treatment of many cases, and more especially in fever cases, are almost indispensable. In Calcutta, where there is a large number of unmarried men whose houses are ill-adapted for cases of sickness the boon of having a hospital to go to where proper treatment and efficient nursing can be secured, is undoubtedly very great. At the Medical College Hospital there was 1 matron, 27 nurses, and 6 dhais employed; at the Eden Hospital there was 1 matron, 3 ward nurses, 2 Canning nurses in training, 12 pupil nurses, 8 ward dhais, and 8 Government pupil dhais; at the General Hospital 25 nurses were employed. Thirteen pupil nurses passed out of the Eden Hospital and obtained diplomas during the year. Of these, seven passed with credit. Eight pupil dhais also passed, and one with credit."

We append a summary of the income and expenditure of the Calcutta Medical Institutions for the two years 1890 and 1891:—

(a) INCOME OF THE CALCUTTA MEDICAL INSTITUTIONS.

| | 1890. | 1891. |
|---|---|---|
| Opening Balance ......... | 15,896 | 16,289 |
| From Government......... | 317,037 | 321,845 |
| Local Funds ................. | 65,443 | 67,527 |
| Municipal Funds ......... | 34,347 | 34,621 |
| Interest on Investments | 24,917 | 27,993 |
| Sale of Securities ......... | — | 4,173 |
| Subscriptions { Europeans | 10,659 | 12,319 |
| Subscriptions { Natives. | 1,512 | 11,696 |
| | 136,878 | 158,329 |
| Total ......Rs. | 469,811 | 496,463 |

(b) EXPENDITURE OF THE CALCUTTA INSTITUTIONS.

| | 1890. | 1891. |
|---|---|---|
| On Establishment ......... | 188,857 | 186,736 |
| On Bazaar Medicines ... | 4,321 | 4,216 |
| On European Medicines | 24,195 | 23,708 |
| On Diet ...................... | 107,493 | 112,221 |
| On Miscellaneous Charges | 61,414 | 75,786 |
| On Buildings and Repairs | 67,242 | 66,776 |
| Invested during the year | — | 10,000 |
| | 453,522 | 479,443 |
| Closing Balance ............ | 16,289 | 17,020 |
| Total ... Rs. | 469,811 | 496,463 |

The average cost for each diet varies, and a full explanation of the figures has been asked for. At the Medical College Hospital the average was 6a. 9¾p. for Europeans, and 3a. 1¾p. for natives. At the Howrah General Hospital the average was 8a. 6p. and 2a. 3p. respectively, while for natives the average cost was 3a. 7p. at the Chandney and Mayo Native Hospitals, 2a. 4p. at the Police Hospital, and 2a. 3½p. at the Campbell. The cost for Europeans at the Presidency General Hospital was 8a. 9p.

In conclusion of this notice of the Calcutta Medical Institutions, and by way of preface to our summary of the reports from the other provinces in the Bengal Presidency, we ought to say that it is only every third year that the reports can be described as full and detailed. In the two intervening years, of which the year under review is one, they are confined to statistics with the briefest possible comments by the Inspector-General. It is, unavoidable, therefore, that our abstract should in this case rather bristle with figures, but if somewhat dry, it none the less offers food for reflection to all who have the interests of our Indian fellow-subjects at heart.

